# Exploring challenges in quality and safety work in nursing homes and home care – a case study as basis for theory development

**DOI:** 10.1186/s12913-020-05149-x

**Published:** 2020-04-03

**Authors:** Terese Johannessen, Eline Ree, Ingunn Aase, Roland Bal, Siri Wiig

**Affiliations:** 1 Faculty of Health Sciences, SHARE - Centre for Resilience in Healthcare, University of Stavanger, Kjell Arholms gate 39, 4021 Stavanger, Norway; 2 Erasmus School of Health Policy & Management, Erasmus University, Rotterdam, The Netherlands

**Keywords:** Nursing home, Home care, Quality challenges, Safety, Leadership, Employees, Context, Theory development

## Abstract

**Background:**

Management, culture and systems for better quality and patient safety in hospitals have been widely studied in Norway. Nursing homes and home care, however have received much less attention. An increasing number of people need health services in nursing homes and at home, and the services are struggling with fragmentation of care, discontinuity and restricted resource availability. The aim of the study was to explore the current challenges in quality and safety work as perceived by managers and employees in nursing homes and home care services.

**Method:**

The study is a multiple explorative case study of two nursing homes and two home care services in Norway. Managers and employees participated in focus groups and individual interviews. The data material was analyzed using directed content analysis guided by the theoretical framework ‘Organizing for Quality’, focusing on the work needed to meet quality and safety challenges.

**Results:**

Challenges in quality and safety work were interrelated and depended on many factors. In addition, they often implied trade-offs for both managers and employees. Managers struggled to maintain continuity of care due to sick leave and continuous external-facilitated change processes. Employees struggled with heavier workloads and fewer resources, resulting in less time with patients and poorer quality of patient care. The increased external pressure affected the possibility to work towards engagement and culture for improvement, and to maintain quality and safety as a collective effort at managerial and employee levels.

**Conclusion:**

Despite contextual differences due to the structure, size, nature and location of the nursing homes and home care services, the challenges were similar across settings. Our study indicates a dualistic contextual dimension. Understanding contextual factors is central for targeting improvement interventions to specific settings. Context is, however, not independent from the work that managers do; it can be and is acted upon in negotiations and interactions to better support managers’ and employees’ work on quality and safety in nursing homes and home care.

## Background

Management, culture and systems for better quality and safety have been the main topics in Norwegian national healthcare policy [1–3]. These key challenges have also been highlighted internationally [4–7]. There is a clear need to ensure leadership, a culture of openness and learning, and a system for developing, embedding and sustaining quality and safety improvements. The delivery of healthcare is becoming more complex as a result of demography, patient preferences and limited resources [4, 6]. A pressure for immediate change may create a cultural bias to jump to implementation without a thorough planning of interventions [4, 6]. These factors can undermine performance and explain some variations in quality and safety [4, 8].

Both Norwegian health policy [1–3] and a recently updated regulation for the entire healthcare services focus on the role of management in quality and safety improvement [9]. The regulation states that management is responsible for the organization to provide professionally sound services and to work systematically with quality improvement and patient and user safety [9]. The regulation also elaborates on the requirements and responsibility for managers to having the overview of quality and safety challenges and risks and to ensure systematic improvement work. Moreover, the regulation specifies the governmental expectations towards healthcare managers for having a quality-oriented management and sound quality management systems in place. Yet, the challenges remain with managers in how to plan, manage and improve healthcare services. Understanding contextual barriers and challenges in quality and safety work in healthcare is crucial to implement effective improvement [7]. The Norwegian healthcare system has increasing knowledge about hospital settings but knows little about how nursing home and home care managers experience quality and safety challenges [2]. Norwegian research by Glette et al. [10] shows that managers and employees experience patients as sicker and more complex in nursing homes and that patient care is also becoming more time-consuming. Specific challenges in home care are the unregulated environment, fragmentation of care, discontinuity and multiple care givers that lack overview of patient status [6, 11]. There are fewer quality indicators in both homecare and nursing home settings in Norway, compared to specialized healthcare services (e.g. hospitals). However, we have seen a development in this area focusing on indicator development such as hospital readmission rates, waiting time for a nursing home placement, waiting time for homecare services, nutrition, competence level (proportion of employees with healthcare education in municipal health care services*)*, dental services last 12 months, hours of doctor per resident in nursing homes, and activities for residents with dementia or disability. Despite these examples of measurable challenges in these settings, we have limited knowledge about healthcare professionals’ own experiences of key challenges. In this paper, we therefore focus on nursing homes and home care as there is a need to map the challenges in quality and safety work, as perceived by managers and employees in Norwegian nursing homes and home care services.

### The SAFE-LEAD project

This paper is part of a larger project titled ‘Improving Quality and Safety in Primary Care – Implementing a Leadership Intervention in Nursing Homes and Home care’ (SAFE-LEAD) [12], based on an intervention implementing a leadership guide for managers over a period of 12 months in 2018–2019 [12]. The leadership guide comprises seven common quality challenges (structure, coordination/organizational politics, culture, competence, engagement, physical design/technology, external demands) in which the organizations work with and diagnose themselves [13, 14]. In this paper, we map the status of the organizations in relation to the seven challenges before the intervention. The aim of this intervention is to build leadership competence and guide managers in improving quality strategies and practice and in tailoring them to their needs [14].

### Aim and research question

The aim of the study was to explore the current challenges in quality and safety work as perceived by managers and employees in nursing homes and home care services before the intervention started. In addition, we were interested in their experience on factors that could facilitate or hinder their quality and safety work.

The following research question guided the study: What are the perceived current challenges in the quality and safety work of managers and employees in nursing homes and home care services?

## Methods

### Study design

The research was conducted as a multiple explorative case study of two nursing homes and two home care services in four municipalities in Norway. The cases allowed for exploration of the differences between nursing homes and home care services and the similarities and differences among municipalities and between managers and employees.

### Case selection and recruitment

The cases were selected based on criteria such as size, geography and variation between city- and rural-based services [12, 14, 15]. Recruitment of the study sites (nursing homes and home care services) was conducted by three nurse-counselors from the Centre for Development of Institutional Care and Home Care Services (USHT). They are employed as co-researchers in the SAFE-LEAD project.

### Context

In Norway, municipalities are responsible for the provision of primary health care services such as nursing homes and home care services, general practitioners (GP) and emergency rooms. Nursing homes provide patients with 24-h stay, treatment and care that requires more health-related work than is practicable and justifiable in the patient’s own home. Nursing homes have different departments such as long-term care, sheltered stay for dementia, and short-term stay. The nursing homes must have access to a nursing home doctor and other relevant professional groups, such as priest and physiotherapists. Home care services coordinate and provide health care services in the patient’s home. The home care assists with tasks such as administration of medicine, personal hygiene, wound and palliative care [16]. Norwegian municipalities have great freedom in the organization and funding of their nursing homes and home care services. This freedom can ensure that municipalities design the services to fit local needs [3], but it results in differences in the delivery of healthcare services. Included in this study were four municipalities and four units; two home care services and two nursing homes. Geographical location was important in selection of units, as well as the different contextual nature between nursing homes and home care services, to explore different challenges they might experience in quality and safety work. The municipalities and units differed in size. Table 1 gives and overview of the study context and a description of the cases.

**Table 1:** Overview of context

Case	Municipality population (approximate N of inhabitants)	Organization	Approximate number of employees	Approximate number of patients	Managerial levels
Home care A	15–20,000 District, medium-sized municipality.	• Delivers home care services • Practical assistance • Responsible for a community based activity center	< 100 Registered nurses Healthcare workers Assistants	280	2
Home care B	5000–10,000 Rural municipality, border to big municipality. Future merging with neighbor municipality.	• Delivers home care services • Practical assistance	< 100 Registered nurses Healthcare workers Assistants	100	2
Nursing home A	130–135,000 Large city, municipality.	Seven departments: • 1 short-term department • 1 drug care department (residence for patients with substance abuse) • 3 dementia departments • 2 long-term departments	200–300 Registered nurses Healthcare workers Assistants	130	2
Nursing home B	70–75,000 City, large municipality in area. Merger with another municipality planned.	One department divided into three groups: • 1 dementia group • 2 long-term groups	< 100 Registered nurses Healthcare workers Assistants	30	2

### Sample

The participants were recruited as a part of a first phase in the SAFE-LEAD project [12] to explore the perceived quality and safety challenges before implementing intervention and as a basis for process evaluation in the project. Each unit selected participants (managers and employees) to participate in the interviews. The total included participants consisted of five males and 31 females. Participants varied in their years of experience as managers and employees. The managerial levels spanned from top managers and unit managers of the nursing homes and home care services, department managers with personnel responsibility for one or several departments within the nursing homes and home care service, one home care coordinator, and two professional development nurses in the nursing homes. Employees ranged from registered nurses to healthcare workers.

### Data collection

Data collection consisted of seven focus group interviews with managers (*n* = 17) and employees (*n* = 19) and two semi-structured interviews with managers in one nursing home (Table 2). The managers in this nursing home were not located in the same unit so individual semi-structured interviews were more convenient. All invited participants consented to participate. No participants declined. The interviews were conducted in March/April 2018. All interviews were based on an interview guide based on the Organizing for Quality framework (OQ) [17] with questions pertaining to *structure, politics, culture, education, emotions, physical and technological factors.* Examples of questions were: What are the key challenges in your quality and safety work? How are you working to create enthusiasm among employees in the quality and safety improvement work (time for it, meeting points, responsibility, opportunity to attend conference, networking, monitoring of results)? How do you as a manager facilitate competence-development among employees? How are decisions on implementation/ changes regarding quality and safety efforts made in this nursing home/home care (needs, motivations, top down, experienced problems in practice)? What is your experience on how data- and information systems support quality and safety improvement? How do you as a manager work with local adaptions on national policies? How do you adjust them to the local unit? Moreover, we asked follow-up questions focusing on challenges, obstacles and facilitators related to each theme.

**Table 2:** Data collection and methods

Case	Method	Informant	Time/duration
Home care A	Focus group interview with managers (1) Focus group with employees (1)	Managers (*n* = 4) Employees (*n* = 4**)**	April 2018/60–90 min
Home care B	Focus group interview with managers (1) Focus group with employees (1)	Managers (*n* = 3) Employees (n = 4).	April 2018/60–90 min
Nursing home A	Focus group interview with managers (1) Focus group with employees (1)	Managers (*n* = 8) Employees (*n* = 6)	April 2018/60–90 min
Nursing home B	Semi structured interviews with managers (2) Focus group with employees (1)	Managers (*n* = 2) Employees (*n* = 5**)**	March 2018/45–90 min
Total	7 focus group interviews 2 semi structured interviews	17 managers 19 employees	

Researchers and co-researchers in the SAFE-LEAD project conducted the interviews in the nursing homes and home care services in which the participants worked. Each interview lasted 60–90 min. All interviews were audio recorded and transcribed verbatim by a professional transcription service. Table 2 gives an overview of data collection and methods.

### Data analysis

The data material was analyzed using directed content analysis approach according to predefined categories [18]. The predefined categories derived from theory, the OQ framework [17] and a further refinement by Johannessen [14]. The categories were structure *(plan and structure for the organizations’ quality work),* coordination and organizational politics *(interaction within the organization and between service levels),* culture *(create an organizational culture where quality is a common value),* competence *(continuous competence development in the organization)*,engagement *(support and mobilize employees to create motivation in the quality improvement work),* physical design and technology *(implies premises, outdoor areas and the importance of home environment for quality improvement work),* and external demands *(awareness and decision related to social, political and economic factors such as regulatory requirements, national professional guidelines).*

Direct content analysis is a deductive approach to interpret meaning from the content of text data. Analysis starts with a theory or relevant research findings as guidance for initial codes. Data were collected followed by questions about the predefined categories. The next step in analysis was to highlight passages using the predetermined codes. Codes were defined before and during data analysis. Text that could not be categorized with the initial coding was identified and analyzed later to determine if they presented a new category or a sub category of an existing code [18]. The OQ framework and a further refinement by Johannessen [14] guided the discussions of findings (17). The main strength of a directed approach to content analysis is that existing theory can be supported and extended. The first author TJ was responsible for the analysis with input from ER and SW who read the transcripts and discussed theme development throughout the analysis period. IA and RB took part in discussion on theme development and refinement. Within-case analysis in each municipality was conducted first, followed by a cross-case analysis to map similarities and differences among municipalities, between nursing homes and home care and between managers and employees.

## Results

The analysis of the data material is presented in two main categories with their associated subcategories. The first category, *structure, coordination, organizational politics, and external demands* describes challenges in continuity, change processes, coordination and how quality systems do not always interact. The second category, *culture, competence and engagement,* describes the challenges related to cultures of errors, maintaining competence among employees and knowledge transfer.

### Structure, coordination, organizational politics, and external demands

#### Keeping continuity is a major challenge affecting service quality and safety

Managers and employees in all units experienced challenges with care continuity. However, these challenges were described in different ways across units, based on variations in the units’ organizational structures. In both home care services, worklists (each employees’ individual list of patients and duties for their specific work shift) were an important part of the quality and safety work. Fixed worklists, where employees attend to the same patients over time, contributed more to care continuity, quality in follow-up, good relationship between patients and personnel, and that decisions were more easily made with patients in their home, than did rotating lists. One of the unit managers expressed the advantages of fixed lists:

The side effect [of the fixed list] is that it is much more fun to work. That everyone should try everything and experience variety in work tasks.… (..) It is when there is case continuity, then you as a nurse or healthcare worker are able to see the changes in the user conditions (Unit manager, home care B).

Employees in the home care services noted that the disadvantages of fixed worklists were less time and opportunity for discussions about ethics. Furthermore, patients were placed on the worklist based on geographical area rather than on employees’ knowledge about the patients. All units shared some challenges in maintaining continuity, such as part-time positions, sick leave, requirements for larger positions, evening adaptation and maternity leaves among the employees. The nursing homes did not have fixed worklists but focused on primary nursing and the ‘primary contact role’, meaning that one employee bore the main responsibility for a group of patients. This ensured more continuity of care and follow-up. The largest nursing home experienced a challenge with many employees working in small positions of 11–12%. Although these part-time employees were expected to follow the same systems as full-time employees, the unit managers found this difficult, as these employees were often not present when the manager was. For example, the managers at nursing home B wanted the ‘primary contact’ to update the care plan regularly in order to have an awareness of the plan and to maintain service quality. Employees working full time had the overview, but the managers worried about keeping the overview during summer vacations and other seasonal changes in staffing and emphasized the importance of preparing for them. Depending on the unit that the managers represented, their perspectives of nursing coverage differed. For example, in home care A, the managers found it important to have enough nurses present. The manager argued that this was less of a problem in nursing homes because one nurse was always present. However, our results showed that even in the large nursing home with several departments, the managers faced similar difficulties with nursing coverage. Sometimes department managers performed nursing duties during the day shift, or one nurse assumed responsibility for approximately 130 patients across seven departments. The following quote illustrates the challenge with nursing coverage:

And if there are several patients who need a nurse then they have to wait. Whether it is just an extra pill they need or if it is a wound to be cleaned. That is noticeable for our patients. (Employee, nursing home A)

#### Managers often have to carry out change processes, adding to the employees’ workload

In several units, the managers described having to balance budget and that this effort sometimes was in conflict with quality and safety work in the organization. Retrenchment in the budgets should not come at the expense of services. The manager in home care A described operational tasks as time consuming, and as taking time away from employees:

Our leeway is reduced. Looking at our efficiency in the home care service, it is incredibly high! We have measured and really made sure that people are so effective that we are at the limit. But, at the same time we have to cut the budget. Therefore, our major challenge is to do things differently, to create room for maneuver. (Unit manager, home care A).

The results showed a contrast among managers and employees in relation to finances and room for improved efficiency. The managers described the need to stick to their budgets and thus relied on trying to establish routines and change processes that met the constant demands for efficiency. For their part, employees reported having to do extra tasks in addition to their regular work. Having to make lunches, order food, and take blood samples without an accompanying increase in resources meant that they had less time to spend with patients and a reduction in the quality of health services.

#### The municipality’s influence on the quality and safety work and the use of quality systems

Managers in the nursing homes reported that one system after another was being imposed upon them. They explained that the municipality bought IT systems, for example a documentation program that was not suited to their needs. The managers found that there were too many systems and too much parallel documentation. Managers in both nursing homes stated that national and municipal political agendas set expectations they had to meet. The manager in home care B, used national plans and guidelines in meetings with the municipal manager to justify their service’s resource needs, by noting the challenges in dementia care and the governmental expectations for service provision to this patient group. In this home care service, resources were reallocated to create a new position for a dementia coordinator, as prescribed in a new national guideline that will be implemented in 2020 (‘dementia 2020’). This manager also talked about major reforms such as the change of care district and a future merger with another municipality. Such decisions were taken at the municipal level without input from the managers in our study. According to the managers in home care B and nursing home B, employees were not interested in the major organizational changes. Employees seemed to be more occupied with their daily tasks and did not mention any concerns about how organizational changes could affect their future work practice. However, employees argued that because of changes introduced at the municipal level, the managers were less visible in the department, causing increased workload and less patient follow-up from management. One unit manager described the effect of change processes on their work:

Is it very much that, a lot of projects and changes, setting up a new group, this is at the expense of how you manage to be available in the workgroup, and how you can try to stay in the forefront yourself (Unit manager, home care A) .

#### Quality systems not interacting and lack of management tools challenges quality and safety work

Employees stated that their municipal quality systems did not communicate with the hospital systems. For example, because of different patient record systems in the municipalities and hospitals, they were not receiving sufficient data about their patients during care transitions. Furthermore, the different systems complicated the training of new employees, especially during the summer vacations. In home care B, employees had online access to the patient record system via tablets. Results showed that they had easy access to patient data as important for quality of care. However, the information from the hospital and GP did not connect with the tablet, so they had to connect from computers in the offices. The shortage of computers was another problem. Managers and employees across all units would have preferred having a laptop or tablet so that they could sit with the patients in their living room when documenting. However, an unreliable wireless network, and poor communication systems between the nursing home and the GP made this impossible. When the quality systems worked as intended, employees and managers found them both helpful and necessary. Both the top manager and the department manager in nursing home B reported that the quality system was efficient and gave them an overview of tasks. In terms of targets, the managers explained that they reported twice a year to the municipal management level about practical tasks such as contract of employment and if patients had been offered individual plans. The reporting seemed to be a safety check. There were variations in the use and need for checklists in the different departments, but what was evident across units and departments was the lack of management tools to guide managers in their quality and safety work. According to one manager:

We are doing a lot of innovation and change processes at the moment, for example running a project now on tightening really, or cutting [resources] in home care. To get some management tools in this [would help]: “How can I be a good manager then?” (Unit manager, home care A).

### Culture, competence and engagement

#### Lack of time affects quality and safety work and leads to different cultures of error reporting

In all units, the most often-reported errors pertained to medication administration and lack of documentation when prescribing medications. Medication errors increased in summertime when more employees were on vacation. Employees talked about the challenges they often faced and that differences in employees’ work culture and work pressure led to poor documentation. The managers acknowledged that this stress easily led to deviations and medication error. At the same time, managers heard complaints directly from patients and relatives who said that employees were not spending enough time with patients and just ran in and out. However, the managers experienced that they had limited capacity to change the situation. One employee described the challenge with lack of documentation when prescribing medications:

We actually had a case here on Tuesday, I think it was, then there was a patient, who had received his medication in the evening, but it had not been signed, and then he believed that he did not get it, and then it was the night shift: but the medicine was not in the medicine trolley. We did not want to give him double dosage. There was no one who knew. So most likely, he got it, but it was not signed and the medicine was gone. (Employee, nursing home A)

Employees in home care B said that they had an inadequate incident reporting practice due to lack of time. One employee said that when all workdays were busy, she was not eager to take the time after her shift to complete the reporting. Other reasons were fear of reporting colleagues, and that the manager would ask why mistakes had been made. The manager in home care B described that it was important to have feedback about errors in order to avoid repeating them. The managers in general wanted to improve service quality and safety and encouraged employees to report adverse events, near misses, and areas they considered as possible improvements. Managers in two small units (home care B and nursing home A) said that when they were away from the units, new cultures quickly formed and that the reporting rate was reduced. At the same time, some managers expressed that employees’ threshold to report was too low. According to one manager, employees were more eager to report different kinds of deviations to explain how busy their morning had been instead of reallocating their resources and getting work done:

You can report deviations on everything. Now I had one example last week ... The one day I was gone, of course when I had given a nurse a leave for half an hour in the morning to accompany her son to a doctor (..) Then they [employees] typically reported, and entered six reports on that day. The patient did not get up at the right time etc. So ... and then we reconsider how much we can anticipate of this? And what is a deviation from good practice? The patient spent half an hour extra in the bed, but it does not necessarily mean that it should be reported. (Department manager, nursing home B)

The top manager in nursing home B said that they had no special routines for processing reports, although they discussed them in the management meetings for learning purposes. Afterwards, the managers addressed them in staff meetings. However, the department manager at the same nursing home stated that there was not enough time during staff meetings to discuss the reports in detail. For their part, employees said that they were tired of hearing about the medications errors and wanted the managers to pay more attention to what they were doing well. They found that, except for medication errors, nothing happened when they reported. The employees also stated a need for more positive feedback and discussion on what went well, as illustrated by the statement from an employee with 18-years experience at the same nursing home:

I have attended those meeting for like 17 to 18 years, and the focus is only on what we can do better! Thus, it is so depressing attending them. They [the managers] are not good at telling us what we do well. Our former manager gave us many compliments, telling us how much we had grown and so on. That means a lot. For so many years, I have thought that I cannot stand more of those meetings, as all they talk about are that we give wrong medicines, this should be better, this is the economy, which is disappointing. The economy has been bad for 20 years. There is no change! (Employee, nursing home B).

Managers and employees did not always know what and where to report. Many systems made reporting difficult. Therefore, managers had trouble disseminating information to all employees in different work positions. The units also differed on how the reports were handled, although managers in all the units found it appropriate to discuss reports frequently. The learning potential was considered the best immediately after an incident was reported, even if the person who had reported was not at work. The latter was also described as a dilemma with shift workers. Other challenges were related to organizing meetings due to sick leave and management being unavailable to follow up.

#### The struggle to maintain competence among employees

Development of competence among the employees was difficult in all four units. Results showed lack of overview of professional specialty among the employees (resource persons). The managers explained how they had tried to map different specialties among employees but struggled to maintain this overview because of constant organizational changes. Common subjects assigned to resource persons were palliative care, hygiene, medicine ordering, and nutrition. Although employees were assigned a subject, there were no results on how they used or maintained this competence. The manager at home care B saw the municipal innovation department as an important support for developing projects and attracting external funding. Moreover, this was explained as an advantage for a small municipality, with very short distances. Managers at all units in our study encouraged employees to take initiative themselves and then offered courses and facilitated development based on this. It was experienced as a strength if employees themselves found an area of ​​interest to elaborate. This was confirmed by the employees. They were eager to take courses that interested them, not just courses that were required for environmental and safety reasons, like the fire course. Several of the managers observed that nurses were more likely to take these courses than other healthcare workers, arguing that the nurses shouldered more of the responsibility for procedures. Employees explained that it was important for assistants to take these courses as well, especially since they often worked full-time positions that affected the overall service quality and competence level in the units. There was also some lack of managerial competence. The top manager at nursing home A acknowledged that many managers in healthcare had been trained as healthcare professionals with limited knowledge of management and leadership. The management role requires different competences than those acquired in nursing education and managers would benefit from more knowledge on management, it was argued.

#### Challenges with knowledge transfer at a formal level due to the healthcare structure

There was a consensus among employees and managers in all units that it was difficult to maintain knowledge transfer at a formal level, especially when employees had been away on courses. Managers thought that employees found it intimidating to stand in front of everyone and share information, so the units rather arranged education and courses themselves. However, employees were kept busy with patient-related tasks and were therefore not able to attend. In addition, employees resented having to come to work on their days off. In one home care, employees believed that a new room with large screen could make 10 min of information sharing easier. The manager in nursing home B ate lunch with employees and employees liked these opportunities for informal discussion. One of the employee described the importance of these informal conversations:

I think we are good at talking together! It is not the same as being taught, but all these conversations are ongoing ... Everything is discussed in the corridors! But the conversations are very informative and they are important for things to go around. (Employee, nursing home B).

## Discussion

Results of this multiple explorative case study showed that the challenges in quality and safety work experienced by nursing home and home care managers and employees had several contributing factors, such as sick leave, work lists, budget cuts, and lack of competence oversight. There were contextual differences in the structure of nursing homes and home care services, although the main challenges in the quality and safety work were common in all units. All managers struggled to maintain continuity of care due to sick leave and constant organizational change processes. This affected the organizational culture and error reporting, especially when the manager was absent.

### The contextual impact on quality and safety work

The context varied in our sample with for example different nursing tasks in home care and nursing homes, differences in size, location, and distance to hospitals. We also found variation in access to reliable networks and communication with GPs in home care and nursing homes that sometimes challenged the staffs and managers work, in line with previous research [19]. However, our results are consistent with previous research showing that one of the greatest leadership challenge is to prepare and facilitate processes for organizational change [20, 21]. During change processes, the managers in our study struggled with the imbalance between available resources and quality and safety work, constantly prioritizing and maneuvering to ensure good practices. In doing so, they adapted their internal contexts (conflicting challenges such as flexible vs. fixed worklist) to fit the external demands. Similar results were reported in a previous study of Norwegian nursing homes and home care services [22]. Furthermore, these findings are in line with research of van de Bovenkamp et al. [23] that uses institutional work when describing how managers both shape and are shaped by their organizational contexts. The increased external pressure reported by participants in our study made it harder for them to strengthen engagement and culture for working on quality and safety, and to maintain this collective effort involving both managers and employees. This also resulted in a lack of oversight of the amount of quality work in the organizations and could have a cumulative negative effect over time [24], due to managers’ struggle to maintain high-quality work. Differences in leadership strategy and in the handling of errors and error reporting were also important in our study, as in other studies [25–28] where employees were demoralized by the constant focus on what was going wrong [29, 30]. Our results indicate that managers and employees should work together more on developing strategies for understanding work practice, challenges and risk and emphasize learning from positive deviance and what goes well [29]. These measures could improve both the organizational learning and work engagement.

The need to make change happened fast in the organizations and the constant struggle to relocate resources and maintain sound services was prominent in our study. This is in line with the research by Katteouw [31] showing that constant external-facilitated reorganization gives professionals less time to do their job. This prioritizes day-to-day operations over the patients’ need for continuity of care. Although managers and employees were constantly trying to improve quality and safety and adapt to external changes, management seemed to lack the tools to create an overview of and plan for quality and safety work. Our results thus support the need of appropriate management tools, despite the increased attention to this at the Norwegian policy level [3]. Our results indicate that a leadership intervention, focusing on giving managers a tool to aid reflection and dialogue to diagnose and take targeted action in their organizations’ quality and safety work, could benefit these participating units. Contextual factors influence quality and safety efforts and their success [32–34]. This study support the importance of the context in these setting. In line with Wiig [35], we suggest targeted contextual factor mapping in nursing homes and home care before and during intervention studies to both tailor the intervention and map the possible influence of contextual factors on the intervention in these settings. Other contextual mapping tools such as the ‘Alberta Context Tool’ are developed to measure context in nursing homes. Our findings fit with several of the factors listed in the tool, such as the challenges related to culture, leadership, social capital, organizational slack, and informal and formal interactions [36–40]. However, our study and research of Ree and colleagues [22] adds to the factors listed in the ‘Alberta Context Tool’ by providing in-depth qualitative descriptions of how the different contextual factors challenges managers and employees’ quality and safety work in these healthcare settings. For example, our study shows how the outer setting, such as external demands from national guidelines, policies and reforms, affects healthcare professionals’ quality and safety work and how they continuously act upon and negotiate the external context to fit local needs. That is, we do not treat context as an independent variable, but something that can at least partly be negotiated by healthcare organizations. A thorough mapping of both inner and outer context is included in the contextual mapping framework by Wiig [35]. The different tools [35–40] can supplement each other when mapping and measuring contextual factors in nursing homes and home care.

### Adaptation of theoretical framework to Norwegian nursing home and home care context

**Fig. 1:**
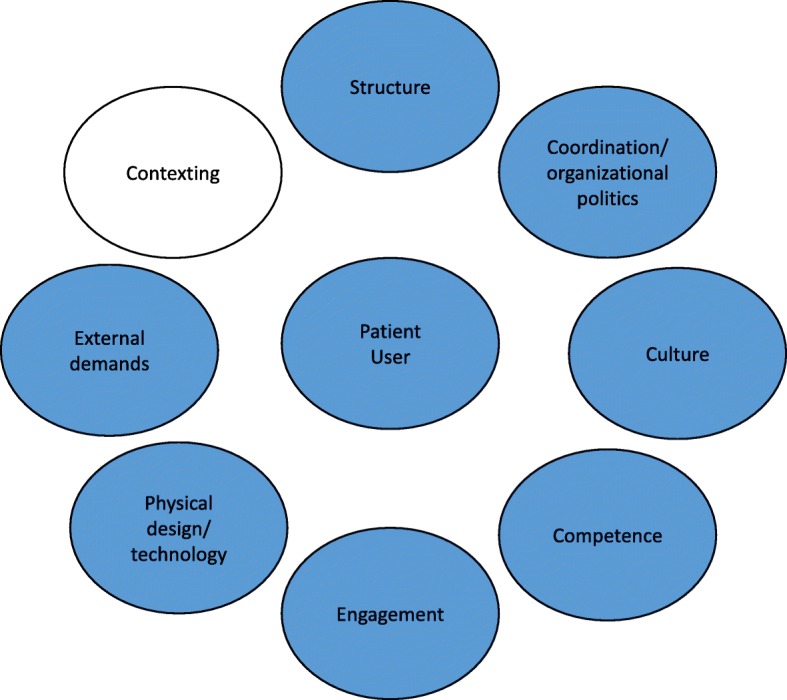
The quality challenges model included the “contexting challenge”. Based on the OQ framework [17] and a further refinement of Johannessen [14]. Generated in power point software. The figure is not under copyright

The OQ model by Bate [17] helps to understand important factors and processes to achieve and maintain high-quality care. Our results demonstrate how external factors such as political decisions, economic pressure, and change processes can undermine quality and safety work, and how they affect internal factors such as collective engagement, competence development and culture. Therefore, our results can be interpreted using the OQ framework to understand which challenges hinder quality and safety work, and how. Furthermore, our results showed a struggle with facilitating and negotiating context. This proved time-consuming for managers and employees alike. The original OQ model defines context as inner and outer context, but context is not conceptualized as a quality challenge. In our studies we have seen the need for adapting the OQ framework into a Norwegian context by using other concepts and revise (Fig. 1) and improve the framework to fit the nursing home and home care setting [14]. Kislov [41] argues for the need to focus on a few key concepts and explore the complex relationships among them, rather than provide exhaustive lists of contextual factors. In our study, we used the concepts of OQ framework while exploring the complex relationships among them. Capturing this complexity in a constantly changing environment requires theory to be constantly refined, and researchers should not rely only on theory to guide research. Focused effort is needed to transparently apply and test existing frameworks [42]. A cross-case study by Bergerød [43] refined the OQ framework based on empirical results to include next-of-kin involvement. It is important to see how the empirical results can be used to refine theory [41]. Hsieh [18] also argues that the strength of a directed approach to content analysis is that theory can be supported and extended. Managers and employees in nursing homes and home care need to incorporate context more actively into their quality and safety work [35] and we argue that our results show a need to refine the OQ theoretical framework applied in our study, by expanding the challenges and adding context as a quality challenge in itself, thereby focusing on ‘context work’. Our study shows the importance of mapping the context in addition to the quality and safety challenges before implementing an intervention to target and direct the intervention to that setting. The additional “contexting” challenge (Fig. 1), indicates that using the OQ framework as a basis for quality and safety improvement work and interventions, implies that context is a challenge that managers, employees and stakeholders need to take into account on a continuous basis and act upon to improve quality and safety. The importance of managers acting upon and negotiating their context as also emphasized in a previous Norwegian study of managers in nursing homes and home care [22].

### Strengths and limitations of the study

The strength of this study is that it contributes with new knowledge to the challenges in quality work in the municipality health care service, and how managers and employees in nursing homes and home care maneuver to continuously change the context in which they work. Given the qualitative nature of this study, the challenges explored were not exhaustive, however they provide insight that could be similar to other nursing homes and home care services [44]. The study has some limitations. First, when using directed content analysis the researcher is more likely to find evidence that supports the theory used. Second, participants could be guided or prompted to answer in certain ways [18]. However, we emphasized that we were interested in all experiences, and that there were no right or wrong answers. Other limitations relate to recruitment of employees, as they were selected by their managers. This could have increased the pressure to participate, although this was necessary to combine interview time with their work schedule and staffing levels. However, all participants were informed about their right to withdraw from the study at any time.

## Conclusion

In this paper, we explored the challenges that managers and employees in Norwegian nursing homes and home care perceived in their work on quality and safety. By using the OQ framework, we identified numerous and sometimes conflicting challenges related to formal and structural elements of the concepts structure, coordination and organizational politics, and external demands, and the softer dimensions of culture, competence and engagement. The interrelated challenges depended on many factors and often implied a trade-off for both managers and employees (budget cut vs. competence development; fixed vs. flexible work lists; learning from errors vs. work engagement; course attendance vs. fear of presenting lessons learnt to others).

There were contextual differences in the structure, size, nature, and location of the nursing homes and home care services, but the challenges were similar across settings. Managers struggled with the upper management in the municipalities that imposed changes that affected their quality and safety work and limited their leeway. Managers struggled to stay visible, available and present in their workgroup; employees struggled with heavier workload and fewer resources that reduced the time spent on and the quality of patient care. The increased external pressure made it harder to work towards engagement and culture for improvement, and to maintain quality and safety as a collective effort at the managerial and employee levels. The findings indicate a lack of tools and limited resources to support managers in balancing the continuous demands for organizational change and establishing a rationale for their priorities during change processes.

The need to understand and act upon contextual factors stood out as crucial. Based on our findings, we have suggested theoretical refinement of the OQ framework by adding “contexting” as a quality challenge (Fig. 1). Our study indicates a dualistic aspect in relation to context. First, understanding contextual factors is central for targeting improvement interventions to specific settings. Second, context can be purposely acted upon in negotiations and interactions to support managers and employees’ work on quality and safety in nursing homes and home care. Further studies should look into the duality of context and how people working in different healthcare settings actively engage with context as part of their effort to improve service provision.

## Data Availability

Original de-identified data of the study will be stored at the Norwegian Centre for Research Data subsequent to completion of the project. Original de-identified data can be requested from the corresponding author upon reasonable request. No database was used in the study.

## Abbreviations

GP: General practitioner
OQ: Organizing for quality

## References

[1] Ministry of Health and Care Services. Meld. St. 10. 2012-2013. God kvalitet trygge tjenester — Kvalitet og pasientsikkerhet i helse- og omsorgstjenesten. Oslo, 2012.

[2] Ministry of Health and Care Services. Meld. St. 11. 2014–2015. Kvalitet og pasientsikkerhet 2013. Oslo, 2014.

[3] Ministry of Health and Care Services. Meld. St. 26. 2014-2015. Fremtidens primærhelsetjeneste – nærhet og helhet. Oslo, 2014.

[4] Dixon-Woods M, McNicol S, Martin G (2012). Ten challenges in improving quality in healthcare: lessons from the Health Foundation's programme evaluations and relevant literature. BMJ Qual Saf.

[5] Vaughn VM, Saint S, Krein SL (2019). Characteristics of healthcare organisations struggling to improve quality: results from a systematic review of qualitative studies. BMJ Qual Saf.

[6] Lindblad M, Flink M, Ekstedt M (2018). Exploring patient safety in Swedish specialised home healthcare: an interview study with multidisciplinary teams and clinical managers. BMJ Open.

[7] Coles E, Wells M, Maxwell M, Harris FM, Anderson J, Gray NM, Milner G, MacGillivray S (2017). The influence of contextual factors on healthcare quality improvement initiatives: what works, for whom and in what setting? Protocol for a realist review. Syst Rev.

[8] Kaplan H, Provost L, Froehle C, et al. The model for understanding success in quality (MUSIQ): building a theory of context in healthcare quality improvement. BMJ Qual Saf. 2012;21:13–20. 10.1136/bmjqs-2011-000010 21835762

[9] Regulation on leadership and quality improvement in the health and care services. Forskrift om ledelse og kvalitetsforbedring i helse -og omsorgstjenesten. Oslo: Ministry of Health and Care Services; 2017.

[10] Glette MK, Røise O, Kringeland T (2018). Nursing home leaders’ and nurses’ experiences of resources, staffing and competence levels and the relation to hospital readmissions – a case study. BMC Health Serv Res.

[11] Lang A, Edwards N, Fleiszer A (2008). Safety in home care: a broadened perspective of patient safety. Int J Qual Health Care.

[12] Wiig S, Ree E, Johannessen T (2018). Improving quality and safety in nursing homes and home care: the study protocol of a mixed methods research design to implement a leadership intervention. BMJ Open.

[13] Anderson JE, Robert G, Nunes F, et al. Translating research on quality improvement in five European countries into a reflective guide for hospital leaders: the ‘QUASER Hospital Guide’. Int J Qual Health Care. 2019;31(8):87–96. 10.1093/intqhc/mzz055 31187862

[14] Johannessen T, Ree E, Strømme T (2019). Designing and pilot testing of a leadership intervention to improve quality and safety in nursing homes and home care (the SAFE-LEAD intervention). BMJ Open.

[15] McDonald KM (2013). Considering context in quality improvement interventions and implementation: concepts, frameworks, and application. Acad Pediatr.

[16] Ringard Å, Sagan A, Sperre Saunes I, Lindahl AK (2013). Norway: health system review. Health Syst Transit.

[17] Bate P, Mendel P, Robert G. Organizing for quality: the improvement journeys of leading hospitals in Europe and the United States. London: Radcliffe Publishing; 2008.

[18] Hsieh HF, Shannon SE (2005). Three approaches to qualitative content analysis. Qual Health Res.

[19] Ko M, Wagner LM, Spetz J (2018). Nursing Home Implementation of Health Information Technology: Review of the Literature Finds Inadequate Investment in Preparation, Infrastructure, and Training. Inquiry.

[20] Yukl G (2006). Leadership in organizations.

[21] Weiner BJ (2009). A theory of organizational readiness for change. Implementation Sci.

[22] Ree E, Johannessen T, Wiig S (2019). How do contextual factors influence quality and safety work in the Norwegian home care and nursing home settings? A qualitative study about managers’ experiences. BMJ Open.

[23] Van de Bovenkamp HM, Stoopendaal A, Bal R (2017). Working with layers: the governance and regulation of healthcare quality in an institutionally layered system. Public Policy Adm.

[24] Vincent C, Amalberti R (2016). Safer healthcare: strategies for the real world.

[25] Glendon I, Clarke S, Mckenna E. Human safety and RISK management. United States: CRC Press; 2006.

[26] Glickman SW, Bagget KA, Krubert CG (2007). Promoting quality: the health-care organization from a management perspective. Int J Qual Health Care.

[27] Wagner LM, Castle NG, Handler SM (2013). Use of HIT for adverse event reporting in nursing homes: barriers and facilitators. Geriatr Nurs.

[28] van Dusseldorp L (2016). Feasibility and added value of executive WalkRounds in long term care in the Netherlands. Jt Comm J Qual Patient Saf.

[29] Hollnagel E, Braithwaite J, Wears RL (2013). Resilient Health Care.

[30] Smaggus A. Safety-I, Safety-II and burnout: how complexity science can help clinician wellness. BMJ Qual. 2019. 10.1136/bmjqs-2018-009147. 10.1136/bmjqs-2018-009147 31196890

[31] Kattouw C, Wiig S (2018). The organisation of community nursing services may impact negatively on safety and the quality of care. Sykepleien Forskning.

[32] Krein SL, Damschroder LJ, Kowalski CP (2010). The influence of organizational context on quality improvement and patient safety efforts in infection prevention: a multi-center qualitative study. Soc Sci Med.

[33] Birken SA, Powell BJ, Presseau J, Kirk MA, Lorencatto F, Gould NJ (2017). Combined use of the consolidated framework for implementation research (CFIR) and the theoretical domains framework (TDF): a systematic review. Implement Sci.

[34] Pfadenhauer LM, Mozygemba K, Gerhardus A, Hofmann B, Booth A, Lysdahl KB (2015). Context and implementation: a concept analysis towards conceptual maturity. Z Evid Fortbild Qual Gesundhwes.

[35] Wiig S, Aase K, Johannessen T (2019). How to deal with context? A context -mapping tool for quality and safety in nursing homes and homecare (SAFE-LEAD context). BMC Res Notes.

[36] Estabrooks CA, Morgan DG, Squires JE (2011). The care unit in nursing home research: evidence in support of a definition. BMC Med Res Methodol.

[37] Estabrooks CA, Squires JE, Cummings GG, Teare GF, Norton PG (2009). Study protocol for the translating research in elder care (TREC): building context—an organizational monitoring program in long-term care project (project one). Implement Sci.

[38] Estabrooks CA, Squires JE, Hayduk L, Morgan D, Cummings GG, Ginsburg L (2015). The influence of organizational context on best practice use by care aides in residential long-term care settings. J Am Med Dir Assoc.

[39] Estabrooks CA, Squires JE, Hayduk LA (2011). Advancing the argument for validity of the Alberta context tool with healthcare aides in residential long-term care. BMC Med Res Methodol.

[40] Estabrooks CA, Squires JE, Cummings GG (2009). Development and assessment of the Alberta context tool. BMC Health Serv Res.

[41] Kislov R (2019). Engaging with theory: from theoretically informed to theoretically informative improvement research. BMJ Qual Saf.

[42] Damschroder, L J. Clarity out of chaos: Use of theory in implementation research. Psychiatry Res. 2020;283:1–6, 112461. 10.1016/j.psychres.2019.06.036 31257020

[43] Bergerød IJ, Gilje B, Braut GS, Wiig S (2018). Next-of-kin involvement in improving hospital cancer care quality and safety – a qualitative cross-case study as basis for theory development. BMC Health Serv Res.

[44] Yin RK (2014). Case study research : design and methods.

